# Direct measurement of electrostatic fields using single Teflon nanoparticle attached to AFM tip

**DOI:** 10.1186/1556-276X-8-519

**Published:** 2013-12-07

**Authors:** Joe-Ming Chang, Wei-Yu Chang, Fu-Rong Chen, Fan-Gang Tseng

**Affiliations:** 1 Institute of NanoEngineering and MicroSystems, Hsinchu 30013, Taiwan; 2 Engineering and System Science Department, National Tsing Hua University, Hsinchu 30013, Taiwan; 3 Applied Science Research Center, Academia Sinica, Taipei 11529, Taiwan

**Keywords:** Electrostatic, Teflon, Nanoparticle, Atomic force microscopy

## Abstract

**Abstract:**

A single 210-nm Teflon nanoparticle (sTNP) was attached to the vertex of a silicon nitride (Si_3_N_4_) atomic force microscope tip and charged via contact electrification. The charged sTNP can then be considered a point charge and used to measure the electrostatic field adjacent to a parallel plate condenser using 30-nm gold/20-nm titanium as electrodes. This technique can provide a measurement resolution of 250/100 nm along the *X-* and *Z*-axes, and the minimum electrostatic force can be measured within 50 pN.

**PACS:**

07.79.Lh, 81.16.-c, 84.37. + q

## Background

Measuring the electrical properties of devices at the micro/nanoscale is an important issue in the semiconductor industry and in materials science [1-4]. Electrical modes in scanning probe microscopy (SPM) [5] have become an essential tool in characterizing the electrical properties at the surface of samples, providing spatial resolution and sensitivity at the micro/nanoscale. Several methods have been developed for the measurement of surface electrical properties and local surface potential, such as electrostatic force microscopy [2,3] and Kelvin probe force microscopy [6,7]. The basic principle behind these techniques [5] is applying a direct current (DC) bias between the conductive probe and the sample to facilitate the recording of variations in the electrostatic force between the probe and sample. These signals are then analyzed in order to interpret the associated surface electrical properties. Jenke et al. [8] used a Pt-coated Si tip with a radius of about 380 nm to probe the electrostatic force generated above embedded nanoelectrodes in the vertical (*Z*) direction. The electrostatic force acting on a grounded conductive tip within an electrostatic field can also be characterized. In this approach, the electrostatic force acting on the atomic force microscopy (AFM) tip comprises Coulombic, induced charge, and image charge forces [9-11]. However, only the Coulombic force is capable of directly revealing the electrical properties of the sample because the two other terms are the result of the AFM tip effect. Kwek et al. [10] glued a charged microparticle to an AFM cantilever to investigate the relative contributions of the Coulombic, induced charge, and image charge forces in the electrostatic force acting on the charged particle; however, the diameter of the charged particle was approximately 105 to 150 μm, which is unsuitable for measurement at the nanoscale.

**Figure 1:**
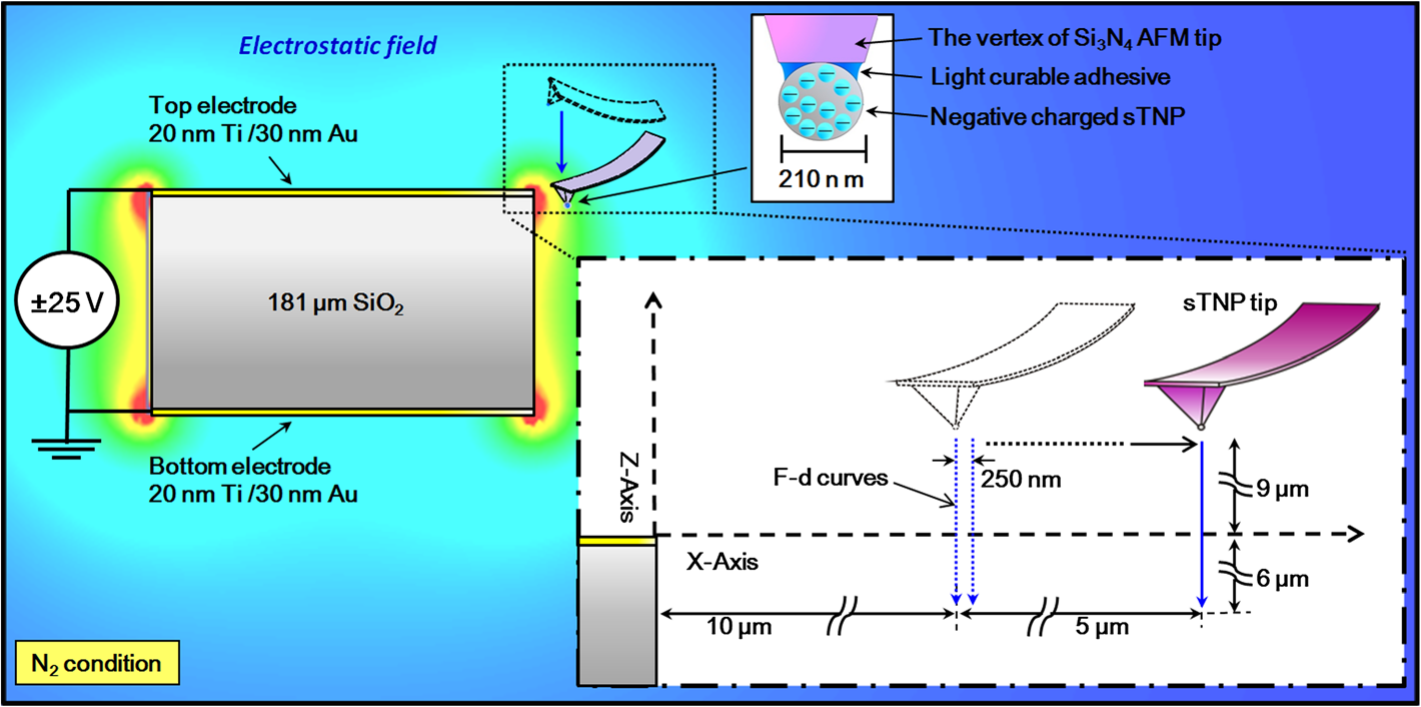
Schematic of experimental setup for the measurement of electrostatic field of a parallel plate condenser.

This paper presents a novel microscopy probe for the direct measurement of electrostatic field (mainly Coulombic force) beside the top electrode of the parallel plate, at a spatial resolution of 250 nm and force resolution of 50 pN(Figure 1). The proposed probe comprises a single 210-nm Teflon nanoparticle (sTNP) attached to the vertex of an insulated Si_3_N_4_ AFM tip (sTNP tip) with charge deposited on the sTNP as an electret via contact electrification [2,12-14]. The parallel plate condenser was fabricated by sputtering layers of Au (30-nm thick) and Ti (20-nm thick) on the top and bottom sides of a 1 × 1 cm glass slide (181 ± 0.25 μm thick). Au was used as the electrode surface and Ti as an adhesion layer. The glass slide was used as the dielectric material. The sTNP tip can be considered a point charge with which to probe the electrostatic force field beside the top electrode of the parallel plate condenser. The electrostatic force acting on the sTNP tip provides direct information related to the local electrostatic field generated in the sample. This technique provides the following advantages: (1) direct measurement of the electrostatic field of a sample is without complex operations or the need for an analytic model, and (2) minimum measurable electrostatic force is within 50 pN with a lateral/vertical spatial resolution of 250/100 nm, respectively.

## Methods

### The process of fabricating the sTNP tip

**Figure 2:**
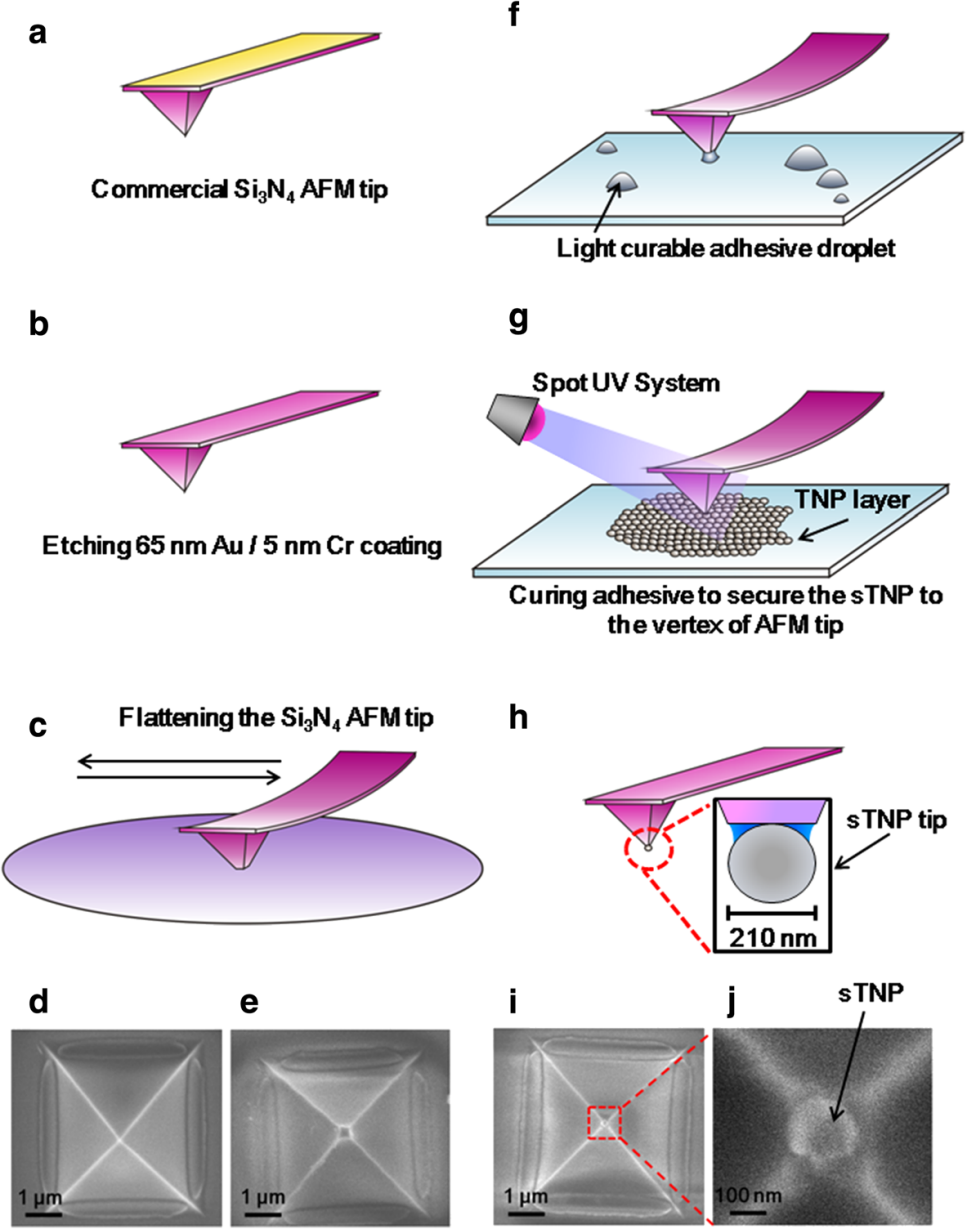
**Schematic diagram showing the process of fabricating the sTNP tip. (a, b)** Etching process for reflective metal layer on Olympus RC-800 Si_3_N_4_ tip. **(c)** The vertex of the tip was flattened by scanning the tip across a polished Si_3_N_4_ wafer. **(d, e)** Present SEM images of the Si_3_N_4_ AFM tip before and after the scanning process, respectively. **(f)** A small quantity of adhesive was applied to the flat top of the AFM tip. **(g)** Attached sTNP to the vertex of the flattened tip with adhesive followed by curing. **(h)** Schematic diagram of fabricated sTNP tip. **(i, j)** SEM images of the sTNP tip.

Figure 2 presents a schematic diagram illustrating the fabrication process of sTNP tip. To obtain insulating Si_3_N_4_ tips for accommodating sTNP, commercial Si_3_N_4_ AFM tips (OMCL-RC800PSA-1, Olympus, Tokyo, Japan) were immersed in gold etchant (Transene, Danvers, MA, USA; 1:1 (*v*/*v*) in H_2_O) for 15 min and in chromium etchant (Cyantek, Fremont, CA, USA; 1:3 (*v*/*v*) in H_2_O) for 40 min to remove the reflective layer of gold (Au) and chromium (Cr) coating the back side of the cantilevers (Figure 2b), respectively. The normal spring constant of the insulating Si_3_N_4_ AFM tip was measured at 0.053 N/m using the thermal noise method [15] with JPK software (JPK Instrument, Berlin, Germany). In order to attach the 210-nm sTNPs, a flat square area with edge length of 300 nm at the vertex of the tip (Figure 2e) was fabricated by scanning a polished silicon nitride wafer (Mustek, Hsinchu, Taiwan) under a large contact loading force of 12 nN at a fast scanning speed of 80 μm/s (Figure 2c). The flattened Si_3_N_4_ AFM tip was cleaned by immersion in a heated (90°C) piranha solution (a 7:3 (*v*/*v*) of 95.5% H_2_SO_4_ and 30% H_2_O_2_) for 30 min. Small droplets of light-curable adhesive (Loctite 3751, Henkel Corp., Way Rocky Hill, CT, USA) several microns in size were spread over the glass slide using a needle. In the application of light-curable adhesive, we employed an inverted optical microscope (IX 71, Olympus) to ensure uniformity in the size of droplets (approximately 5 μm) on the scale of the base length (approximately 4.5 μm) of the pyramidal AFM tip. The cleaned Si_3_N_4_ AFM tip was then mounted on the NanoWizard AFM scanner (JPK Instrument) and brought into contact with the adhesive droplet (Figure 2f). This allowed the placement of a small quantity of adhesive on the flat top of the AFM tip. The tip was then put into contact with the TNP layer deposited on the glass slide (Figure 2g). The TNP layer was prepared by drying a 30-μl droplet (200 nm in diameter) of 5% polytetrafluoroethylene (PTFE) aqueous dispersion (Teflon PTFE TE-3893, DuPont, Wilmington, DE, USA) on the glass slide. PTFE has been shown to possess excellent performance characteristics with regard to charge storage and is widely used in electret applications [16]. The adhesive was cured by exposure to UV radiation illuminated from a spot UV system (Aicure ANUP 5252 L, Panasonic, Osaka, Japan) at 3,000 mW/cm^2^ for 3 min to secure the sTNP. Figure 2d,e presents typical images from a scanning electron microscope (SEM) showing the top views of the Si_3_N_4_ AFM tip before and after the flattening procedure. Figure 2i presents an SEM image of the sTNP tip. The diameter of the sTNP attached to the vertex of the Si3N4 AFM tip was measured by SEM at 210 nm (Figure 2j).

### The experimental setup of the deposition of charge to the sTNP tip

**Figure 3:**
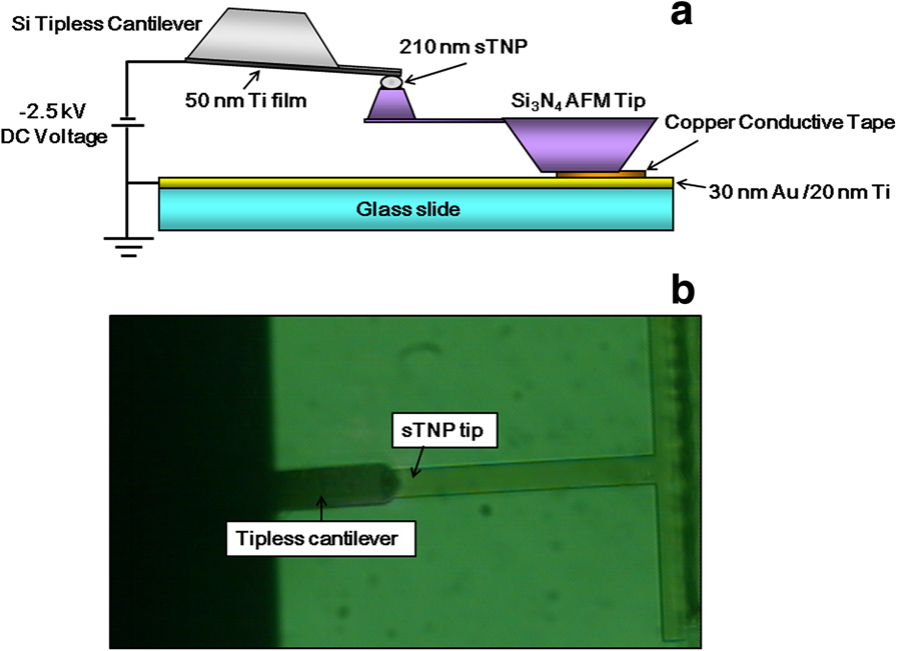
**Schematic diagram of experimental setup for the deposition of charge to the sTNP tip. (a)** Schematic diagram of experimental setup for the deposition of charge to the sTNP at the vertex of the Si_3_N_4_ AFM tip and **(b)** × 40 optical microscope image of the charging setup.

The experimental setup used for the deposition of charge to the sTNP tip is presented in Figure 3. The back side of the sTNP tip was affixed to the 30-nm Au/ 20-nm Ti-coated glass slide using conductive copper tape (3 M, St. Paul, MN, USA). A 50-nm Ti-coated tipless cantilever (CSC12, MikroMasch, Tallinn, Estonia) was mounted on the JPK AFM scanner as the top electrode. The end of the tipless cantilever was positioned precisely on the sTNP at the vertex of the Si_3_N_4_ tip by aligning the JPK AFM scanner under an inverted optical microscope (IX 71, Olympus; Figure 3b). DC voltage (−2.5 kV) was applied to the tipless cantilever for 90 s under air, and the 30-nm Au/20-nm Ti-coated glass slide was used as the ground for the deposition of the negative charge to the sTNP tip. The force-distance (f-d) curves of the sTNP tip on the grounded gold surface were used to verify whether the charge was deposited [17].

### Measurement of the electrostatic fields

The charged sTNP tip was then used for the measurement of f-d curves to determine the electrostatic field beside the top electrode of the parallel plate condenser (Figure 1). The sTNP tip is located slightly inward at the end of the AFM cantilever; therefore, the end of the AFM cantilever is susceptible to striking the edge of the top electrode when the distance between the AFM tip and the electrode is within 10 μm. To overcome this situation, 21 spots spaced at 0.25 μm along the *X*-axis at a distance of 10 to 15 μm are selected for the measurement of the f-d curves in order to derive the electrostatic field. As shown in Figure 1, the edge center of the condenser was plotted as the origin of the *X*- and *Z*-axes. DC voltage (*V*_app_) of ±25 V was applied on the top electrode, and the bottom electrode was left grounded. Each curve measurement was conducted for distances of 15 μm along the *Z*-axis, from 6 μm below to 9 μm above the top electrode. The ramp rate and the ramp size of each f-d curve were 2 Hz and 15 μm, respectively. The sTNP tip did not come into contact with the substrate during the measurement of electrostatic fields. The measurement of f-d curves was conducted using the force mapping function in the JPK SPM software.

### Simulation of the electrostatic field

The electric field was simulated using finite element method in Ansoft Maxwell simulation software [18] to estimate the electrostatic field. The current model deals only with the electric field in the *Z* direction from −10 to approximately 10 μm. After designing the model, the maximum length of elements was set at 0.4 μm; this was sufficient to provide accurate solutions to model at that scale. The Maxwell program automatically fits the mesh to estimate the electrostatic field.

## Results and discussion

**Figure 4:**
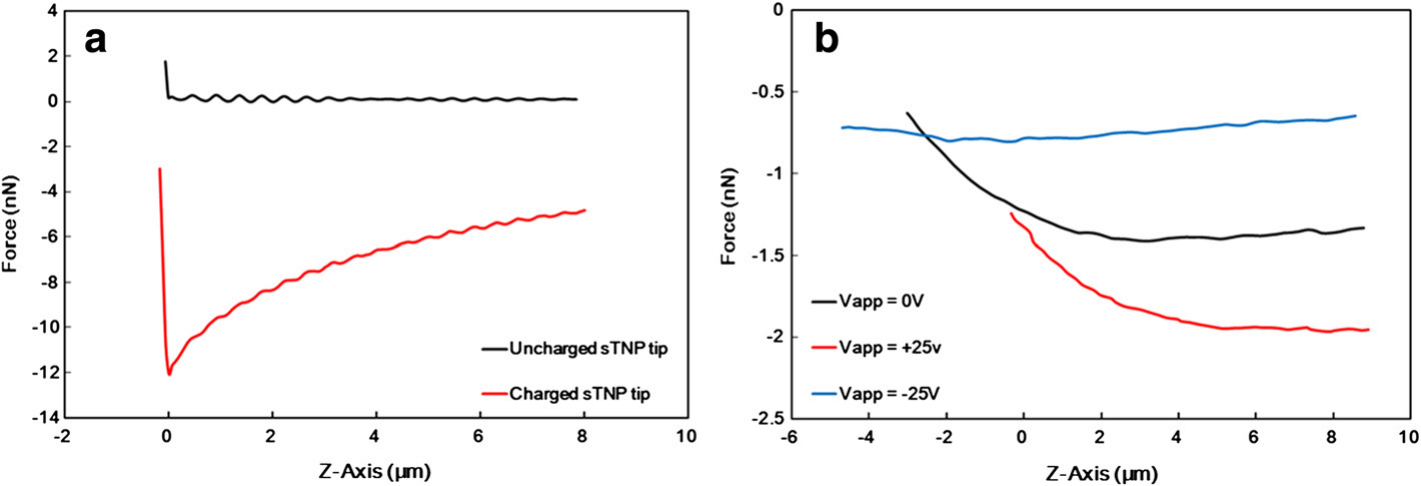
**Schematic diagram of f-d curves conducted using sTNP tip. (a)** f-d Curves obtained from a grounded metal surface using charged/uncharged sTNP tip. **(b)** Electrostatic force acting on charged sTNP tip when *V*_app_ = +25, 0, and −25 V in the *Z* direction at *X* = 11 μm.

Figure 4a presents the f-d curves for tips before and after the charging process. A long-range attractive force [19] was observed between the charged sTNP tip and the grounded gold surface, mainly due to the electrostatic force. No attractive force was observed on the uncharged sTNP tip. The attractive force acting on the charged sTNP tip gradually increased as the tip was moved closer to the gold-coated surface. As shown in Figure 4a, the form of the f-d curve acting on the grounded metal surface using a charged sTNP is similar to that observed in a previous study involving the measurement of electrostatic force between a charged particle and a metal surface using the modified image charge method [17].

According to previous studies [9-11], the net electrostatic force (*F*_E_) acting on a charged dielectric particle in an applied electric field that can be written as follows:

(1) $$F_{E}=F_{C}+F_{\mathrm{image}}+F_{\mathrm{pol}},$$

where *F*_C_ is the Coulombic force that resulted from the external field acting on the charged particle, *F*_image_ is the image force caused by the attraction of the particle to its net charge image, and *F*_pol_ is the force created by the attraction between the field-induced dipolar charge (polarization) in a particle in an electrostatic field and its dipole image in the electrode.

In this study, *F*_pol_ acting on the sTNP was due mainly to the thin layer of water adsorbed on the surface of the tip due to the large dielectric constant of water (*ϵ*_water_ = 80). To eliminate the influence of the water layer, the measurement of the electrostatic field was conducted under N_2_ conditions (RH < 5%), such that *F*_pol_ acting on the sTNP could be disregarded; a plastic O-ring was placed between the scanner and sample to allow the injection of N_2_ into the O-ring. Charges deposited on the sTNP under N_2_ conditions can last (variation smaller than 5%) for over 90 min, and the measurement process can be completed within 10 min. In this study, the dissipation or generation of charge did not occur during charge verification, despite the fact that the charged sTNP tip touched the grounded metal surface under N_2_ conditions. Figure 4b presents the three f-d curves at *X* = 11 μm under N_2_ conditions when *V*_app_ = +25, 0, and −25 V were applied to the top electrode, and the bottom electrode remained grounded. The *Z*-axis component of *F*_E_ acting on the sTNP tip can be revealed in the measured f-d curves (Figure 4b), expressed as *F*_E_(*V*_app_). *F*_E_(0 V) acting on the sTNP tip is due mainly to *F*_image_, which is always attractive to the top electrode of the condenser. The *F*_C_(+25 V) is the attractive force acting on the negative-charged sTNP tip, such that *F*_E_(+25 V) is smaller than *F*_E_(0 V) above *Z* = 0 μm. *F*_C_(+25 V) always attracts the negative-charged sTNP tip, regardless of whether the sTNP tip is above or below the top electrode at *Z* = 0 μm. This results in the charged sTNP tip being trapped at *Z* = 0 μm, preventing it from moving forward during the measurement of the f-d curves, as shown in Figure 4b. *F*_C_(−25 V) is a repulsive force acting on the negative-charged sTNP tip, such that *F*_E_(−25 V) is larger than *F*_E_(0 V) above *Z* = −2.6 μm; however, it is smaller below *Z* = −2.6 μm due to the attractive force induced from the bottom electrode.

**Figure 5:**
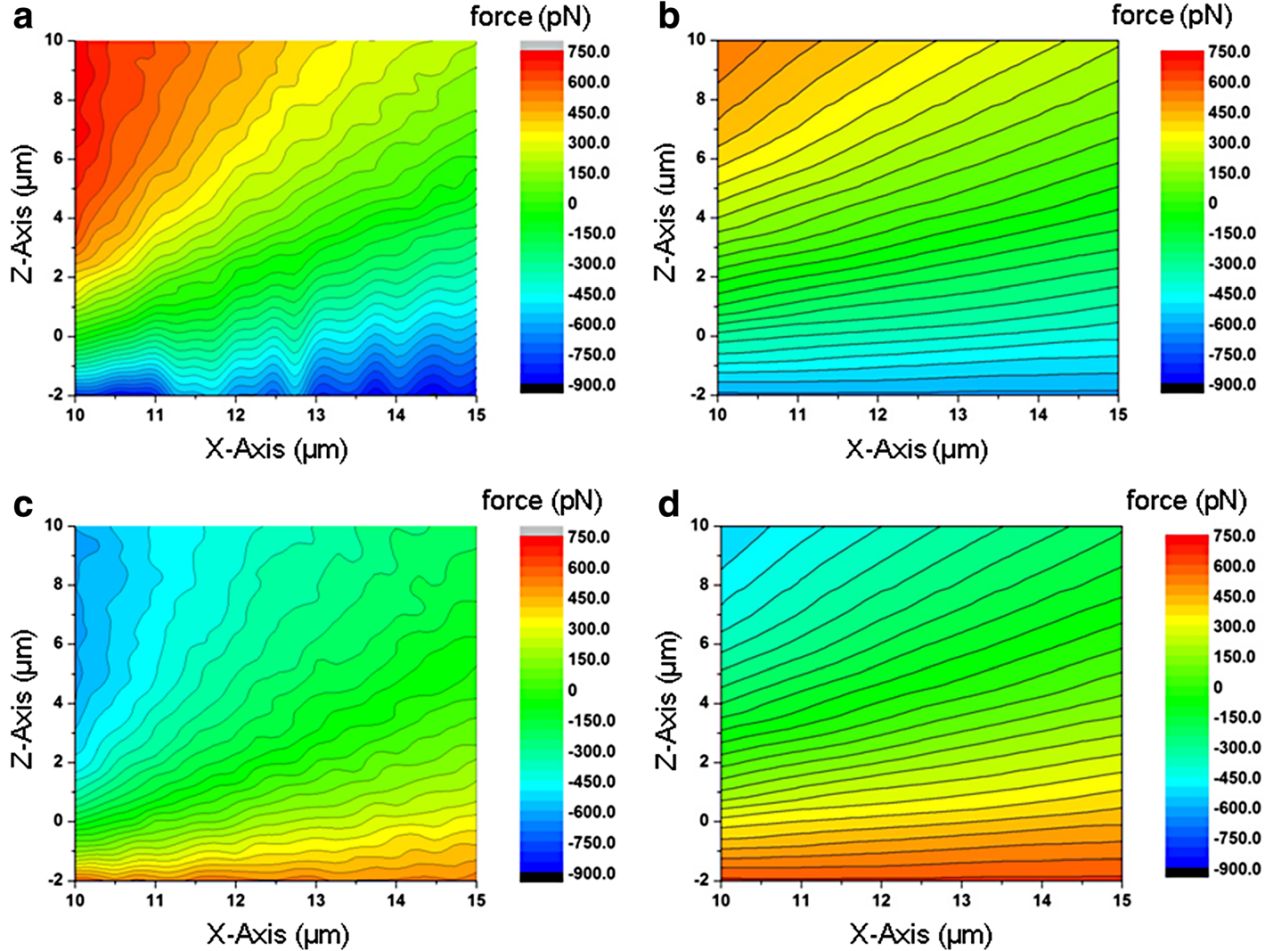
**Experimental results vs. Ansoft Maxwell simulation. (a, c)** The *F*_ele_(+25 V) and *F*_ele_(−25 V) distribution along the *X*-axis (0.25-μm spacing from 10 to 15 μm) and the *Z*-axis. **(b, d)** The results of Ansoft Maxwell simulation of electrostatic field distribution under *V*_app_ = +25 and −25 V, respectively.

Thus, *F*_C_(V_app_) acting on the negative-charged sTNP tip can be estimated according to the following formula: *F*C(*V*_app_) = *F*_E_(*V*_app_) − *F*_E_(0 V). The coulombic force acting on the positive charged sTNP produced by the electrostatic field of the parallel plate condenser is equal to − *F*_C_(*V*_app_), expressed as *F*_ele_(*V*_app_), which represents the electrostatic force field of the condenser. Figure 5a,c respectively presents the *F*_ele_(+25 V) and *F*_ele_(−25 V) distribution along the *X*-axis (0.25-μm spacing from 10 to 15 μm) and the *Z*-axis. As mention in previous discussion, *F*_ele_(+25 V) below *Z* = 0 μm cannot be measured but can be acquired through polynomial extrapolation. In this study, charge was deposited on the sTNP, a small portion of which was transferred to the edge of the pyramid shaped Si_3_N_4_ tip. As a result, the total charge on the sTNP was assumed to be a point charge located 2 μm above the vertex of the Si_3_N_4_ tip. The *Z*-axis in Figure 5a,c reveals the distance between the point charge and the top electrode in the *Z* direction. Figure 5b,d presents the results of Ansoft Maxwell simulation of electrostatic field distribution under *V*_app_ = +25 and −25 V, with trends similar to those in Figure 5a,c, respectively. The charge on the charged sTNP tip was approximately −1.7 × 10^−14^C, as estimated through simulation. *F*_ele_(−25 V) is the attractive force above *Z* = 0 μm; however, this was converted into a repulsive force between *Z* = 0 and −2 μm. *F*_ele_(+25 V) and *F*_ele_(−25 V) are symmetrical about the *Z*-axis, revealing the inverse direction of the electrostatic field distribution. As shown in Figure 5a,c, the minimum *F*_ele_ that can be measured is less than 50 pN. The difference between the simulated and experimental results can be explained by the fact that a residual charge is transferred to the edge of the pyramid-shaped Si_3_N_4_ tip during charge deposition process; when the charged sTNP tip is brought closer to the top electrode (*X* = 0 μm), the difference between the simulated and experimental results increases. The ripples shown in Figure 5a,c were caused by laser diffraction on the insulating Si_3_N_4_ cantilever (for more details, see Additional file 1).

In the future, the pyramidal shape of the Si_3_N_4_ tip could be modified using a focused ion beam system to create a cylindrical shape in order to avoid the possibility of experimental fluctuations resulting from the shape of the tip. This probe could be employed to scan surface topographies by mapping f-d curves, and the interaction force between the charged Teflon particle and sample would give a direct indication of the local electric field and properties of the sample.

## Conclusions

In summary, this paper reported the direct measurement of the electrostatic field beside a parallel plate condenser using a charged sTNP on an AFM tip. Experimental results were then compared with those obtained through simulation. A sTNP tip was fabricated by attaching a single 210-nm Teflon nanoparticle at the vertex of a Si_3_N_4_ AFM tip and was charged via contact electrification. The lateral/vertical resolution of the electrostatic force measurement is 250/100 nm, respectively. The minimum *F*_ele_ that can be measured using this method is less than 50 pN. This technique provides a novel means of studying the electric properties of electrical devices. The AFM tip is able to hold a single charged nanoparticle, making it possible to directly quantify the local electric/magnetic field, charge distribution, and electrostatic force of a sample surface using an AFM system. The charged sTNP tip could find a wide application in electrical research at the nanoscale.

## Abbreviations

AFM: Atomic force microscopy; FC(Vapp): Coulombic force resulting from the external field acting on the charged sTNP tip; FE(Vapp): *Z*-axis component of net electrostatic force acting on the sTNP tip; Fele(Vapp): Electrostatic force field of the condenser; PTFE: Polytetrafluoroethylene; SEM: Scanning electron microscope; Si3N4: Silicon nitride; SPM: Scanning probe microscopy; sTNP: Single Teflon nanoparticle; sTNP tip: Single 210-nm Teflon nanoparticle attached to the vertex of an insulated Si_3_N_4_ AFM tip; Vapp: DC voltage applying on the top electrode.

## Competing interests

The authors declare that they have no competing interests.

## Authors’ contributions

JMC performed all the AFM measurements and wrote the manuscript. WYC carried out the Ansoft Maxwell simulation. FRC provided valuable discussions and helped in Ansoft Maxwell simulation. FGT is the principal investigator who helped in the analysis and interpretation of data and in drafting of the manuscript and its revisions. All authors read and approved the final manuscript.

## Authors’ information

JMC received his M.S. degree in engineering and system science from National Tsing Hua University, Hsinchu, Taiwan in 2005. He is currently working towards finishing his Ph.D. at the Institute of NanoEngineering and Microsystems, National Tsing Hua University, Hsinchu, Taiwan. WYC is currently working towards finishing a Ph.D. degree at the Department of Engineering and System Science, National Tsing Hua University, Hsinchu, Taiwan. FRC is a professor at the Department of Engineering and System Science, National Tsing Hua University, Hsinchu, Taiwan. FGT is a professor at the Department of Engineering and System Science, National TsingHua University, Hsinchu, Taiwan. He received his Ph.D. degree in mechanical engineering from the University of California, Los Angeles (UCLA), under the supervision of Prof. C-M Ho and C-J Kim in 1998. He is currently the Deputy Director of Biomedical Technology Research Center of NTHU and Chairman of the ESS department. He has written five book chapters, including ‘Micro droplet generators’ in *MEMS Handbook* (CRC) and ‘Technological aspects of protein microarrays and nanoarrays’ in *Protein Microarrays* (Jones and Bartlett), and he has published more than 80 SCI Journal papers and 240 conference technical papers in MEMS, bio-N/MEMS, and micro/nanofluidic-related fields. He has received 32 patents. FGT is a member of ASME, APS, and ACS. He has received several awards, including the Mr. Wu, Da-Yo Memorial Award from National Science Council, Taiwan (2005–2008), five best paper/poster awards (1991, 2003, 2004, 2005, and 2009), NTHU new faculty research award (2002), NTHU outstanding teaching award (2002), NTHU academic booster award (2001), and NSC research award (2000).

## Supplementary Material

Additional file 1 **f-d Curves, duration time, and schematic diagram. Figure S1.** f-d curves obtained from a grounded metal surface before and after the measurement of the electrostatic field. **Figure S2.** the duration time of the charged sTNP tip under N_2_condition. **Figure S3.** f-d curves obtained from sTNP tip under N_2_ condition. **Figure S4.** schematic diagram of differences between experimental result and Ansoft Maxwell simulation. (Difference = *F*_ele_ measured by EXP − *F*_ele_ simulated by Ansoft Maxwell). Click here for file

## References

[B1] Martin Y Williams CCHK Wickramasinghe HK Atomic force microscope-force mapping and profiling on a sub 100-A scale J Appl Phys 1987 8 4723 4729 10.1063/1.338807

[B2] Stern JE Terris BD Mamin HJ Rugar D Deposition and imaging of localized charge on insulator surfaces using a force microscope Appl Phys Lett 1988 8 2717 2719 10.1063/1.100162

[B3] Terris BD Sterna JE Rugar D Mamin HJ Localized charge force microscopy J Vac Sci Technol 1990 8 374 377

[B4] Berger R Butt HJ Retschke MB Weber SAL Electrical modes in scanning probe microscopy Macromol Rapid Commun 2009 8 1167 1178 10.1002/marc.200900220 21638372

[B5] Bonnell DA Electrostatic and magnetic force microscopy Scanning Probe Microscopy and Spectroscopy 2001 New York: Wiley 207 210

[B6] Nonnenmacher M O’Boyle MP Wickramasinghe HK Kelvin probe force microscopy Appl Phys Lett 1991 8 2921 2923 10.1063/1.105227

[B7] Palermo V Palma M Samori P Electronic characterization of organic thin films by Kelvin probe force microscopy Adv Mater 2006 8 145 164 10.1002/adma.200501394

[B8] Jenke MG Santschi C Hoffmann P Two-dimensional electrostatic force field measurements with simultaneous topography measurement on embedded interdigitated nanoelectrodes using a force distance curve based method Appl Phys Lett 2008 8 063113 10.1063/1.2844882

[B9] Feng JQ Hays DA Relative importance of electrostatic forces on powder particles Powder Technol 2003 8 65 75

[B10] Kwek JW Vakararelski IU Ng WK Heng JYY Tan RBH Novel parallel plate condenser for single particle electrostatic force measurements in atomic force microscope Colloids Surf Physicochem Eng Aspects 2011 8 206 212 10.1016/j.colsurfa.2011.06.008

[B11] Harris B The electric field University Physics 1995 New York: John Wily & Sons, Inc 455 475

[B12] Terris BD Stern JE Rugar D Mamin HJ Contact electrification using force microscopy Phys Rev Lett 1989 8 2669 2672 10.1103/PhysRevLett.63.2669 10040956

[B13] Mesquida P Stemmer A Attaching silica nanoparticles from suspension onto surface charge patterns generated by a conductive atomic force microscope tip Adv Mater 2001 8 1395 1398 10.1002/1521-4095(200109)13:18<1395::AID-ADMA1395>3.0.CO;2-0

[B14] Mesquida P Knapp HF Stemmer A Charge writing on the nanometre scale in a fluorocarbon film Surf Interface Anal 2002 8 159 162 10.1002/sia.1181

[B15] Hutter JL Bechhoefer J Calibration of atomic force microscope tips Rev Sci Instrum 1993 8 1868 1873 10.1063/1.1143970

[B16] Kestelman VN Pinchuk LS Goldade VA Electrets Engineering: Fundamentals and Applications 2000 Boston: Kluwer Academic Publishers

[B17] Matsuyama T Ohtsuka M Yamamoto H Measurement of force curve due to electrostatic charge on a single particle using atomic force microscope KONA Powder Particle J 2008 8 238 245

[B18] ANSYS, Inc ANSYS Maxwell http://www.ansys.com/Products/Simulation+Technology/Electromagnetics/Electromechanical/ANSYS+Maxwell

[B19] Israelachvili JN Contrasts between intermolecular, interparticle and intersurface forces Intermolecular and Surface Forces 1991 San Diego: Academic 152 155

