# Supplementary material for: Direct measurement of electrostatic fields using single Teflon nanoparticle attached to AFM tip
Source: Nanoscale Res Lett. 2013 Dec 7;8(1):519. doi: 10.1186/1556-276X-8-519 (PMC4029376; doi:10.1186/1556-276X-8-519)
Supplement: Additional file 1 — f-d Curves, duration time, and schematic diagram. Figure S1. f-d curves obtained from a grounded metal surface before and after the measurement of the electrostatic field. Figure S2. the duration time of the charged sTNP tip under N2condition. Figure S3. f-d curves obtained from sTNP tip under N2 condition. Figure S4. schematic diagram of differences between experimental result and Ansoft Maxwell simulation. (Difference = Fele measured by EXP − Fele simulated by Ansoft Maxwell). [file 1556-276X-8-519-S1.pdf]

1. The consistent of the charged sTNP tip :

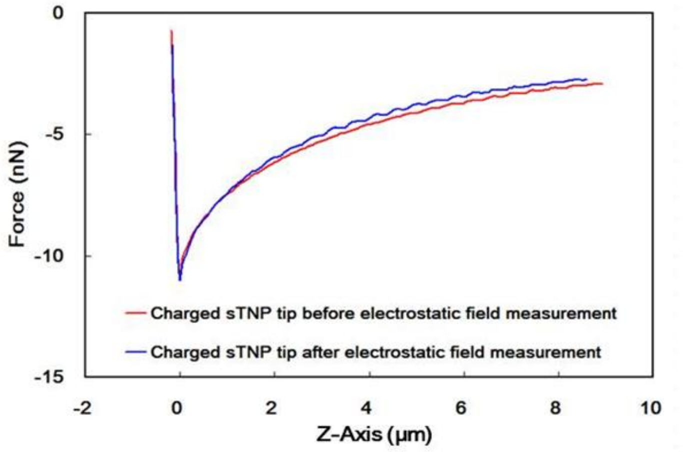

Figure 1 F-d curves obtained from a grounded metal surface before and after the measurement of the electrostatic field

In this study, f-d curves of the sTNP tip on the grounded gold surface were used to verify whether the charge was deposited. An attractive force was observed to be acting on the sTNP tip (as shown on Fig.1), indicating the the sTNP tip was charged.

The attractive force (F_{image}) acting on the charged sTNP tip before and after measurement was -10.82 nN and -10.97 nN, respectively. These results indicate that the deposited charge remained consistent throughout the measurement of the electrostatic field.

The charge deposited on the sTNP exceeded charge dissipation due to moisture during the charging process. Charging the sTNP tip under ambient conditions for a period of 90 s was sufficient for the measurements in this study (Fig.1).

2. The duration time of the charged sTNP tip under N_2 condition :

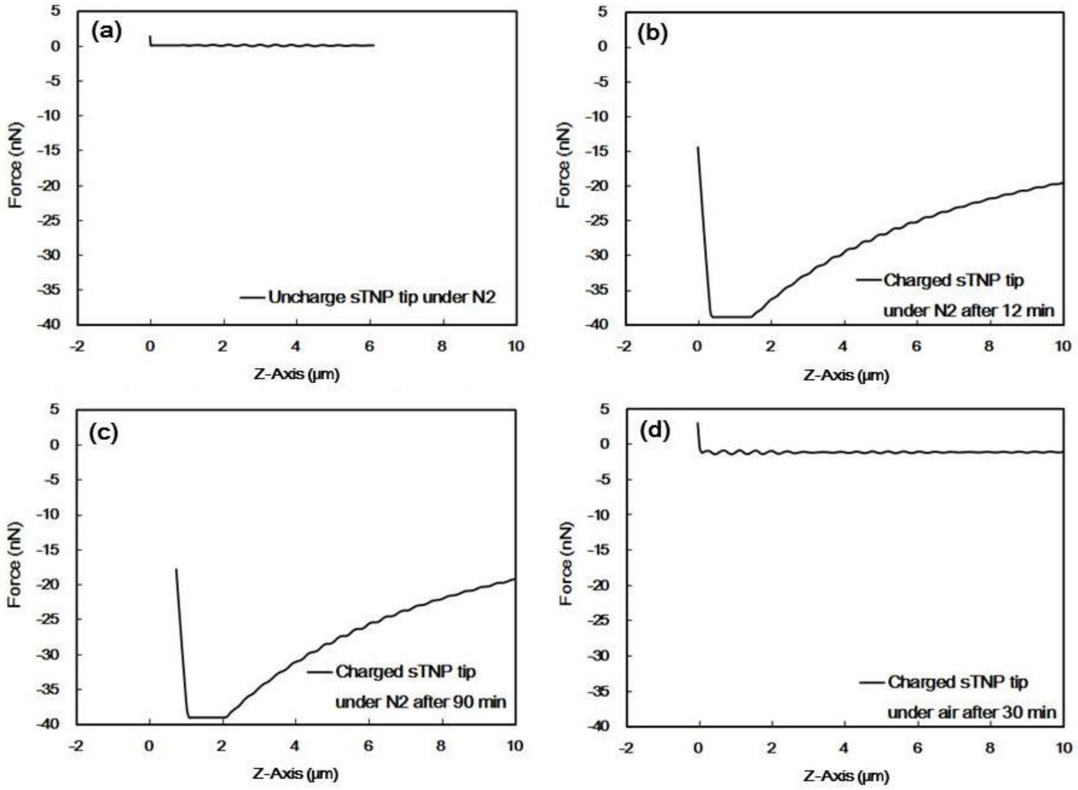

Figure 2 The duration time of the charged sTNP tip under N_2 condition.

(a) F-d curves obtained from a grounded metal surface using an uncharged sTNP tip under N_2; (b) and (c) f-d curves obtained from a charged sTNP under N2 after 12 and 90 min, respectively; (d) f-d curves obtained from charged sTNP under air after 30 min.

Fig. 2 presents the charging durations of the sTNP tip under N_2. Fig. 2(a) presents the F-d curves obtained from a grounded metal surface using an uncharged sTNP tip under N_2. No attractive force (F_{image}) was observed on the uncharged sTNP tip. Figs. 2(b) and (c) present the f-d curves obtained from charged sTNP tips under N_2 after 12 and 90 min, respectively. The F_{image} acting on the sTNP tip is consistent with the results presented in Figs. 2(b) and (c), demonstrating that the charge deposited on a sTNP tip can last for over 90 min. A small portion of the f-d curves was outside the detection range of the photodetector.

Fig. 2(d) presents the f-d curves obtained from a charged sTNP under air after 30 min. The deposited charge dissipated through exposure to moisture and no F_{image} was observed on the charged sTNP tip.

3. The measurement of the F_{pol} :

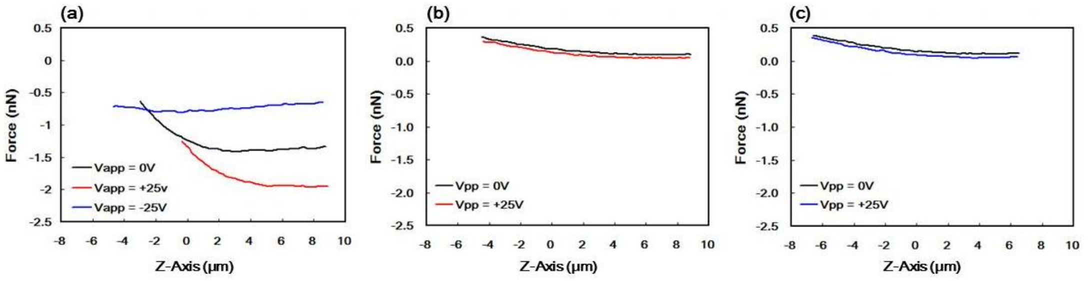

Figure 3 f-d curves obtained from sTNP tip under N_2

(a) Electrostatic force acting on charged sTNP tip when V_{app} = +25, 0, and -25V in the Z-direction at X =11 μm; (b) and (c) present the two f-d curves obtained from the uncharged sTNP tip at X = 11 μm when V_{app} = +25/ 0 V and V_{app} = -25/ 0 V, respectively.

Fig. 3(a) presents the electrostatic force acting on a charged sTNP tip when V_{app} = +25, 0 and -25 V in the Z-direction at X =11 μm. Figs. 3(b) and (c) respectively present the two f-d curves obtained from an uncharged sTNP tip at X =11 μm when V_{app} = +25/ 0V and V_{app} = -25/ 0V under N_2 conditions. F_E(+25V) and F_E(-25V) acting on the uncharged sTNP tip under N_2 conditions are due mainly to polarized charges in the uncharged sTNP tip (F_{image} and Fc = 0). The force (F_{pol}) induced by polarized charges can be estimated as follow:

$$F_{pol}(+25V) = F_E(+25V) - F_E(0V) \quad (1)$$

$$F_{pol}(-25V) = F_E(-25V) - F_E(0V) \quad (2)$$

The F_{pol} (+25V) was -49.41 ± 6.2 pN and F_{pol} (-25V) was -51.41 ± 8.9 pN. The contribution of the polarized charges (~ -50 pN) to the net electrostatic force FE (~500 pN) in Fig. 3(a)) was quite small (~10%).

4. **The differences between the experimental result and Ansoft Maxwell simulation in the in the region X = 10 to 11 micrometers, Z = 4 to 10 micrometers :**

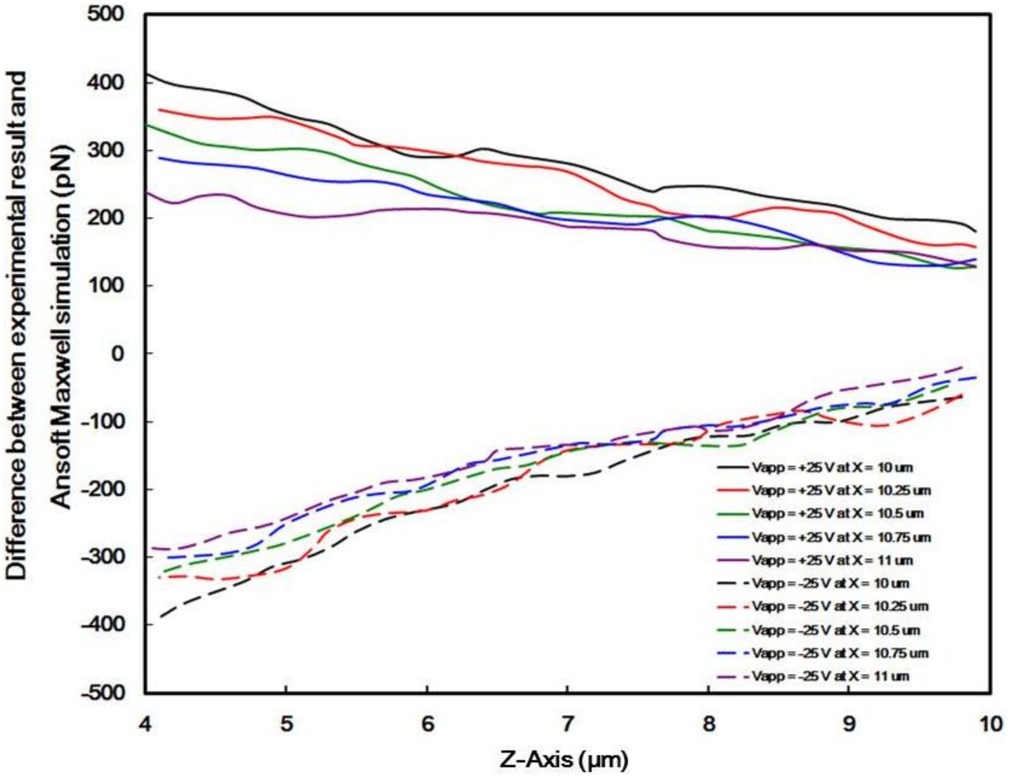

Vapp = +25 V	AVG of difference (pN)	STDV
At Z = 10 micrometers	147.04	22.12
At Z = 4 micrometers	327.99	67.06

Vapp = -25 V	AVG of difference (pN)	STDV
At Z = 10 micrometers	-45.22	18.08
At Z = 4 micrometers	-325.64	39.49

Figure 4 Schematic diagram of differences between experimental result and Ansoft Maxwell simulation. (Difference = F_{ele} measured by EXP - F_{ele} simulated by Ansoft Maxwell)

Fig. 4 presents the differences (Difference = F_{ele} measured by EXP - F_{ele} simulated by Ansoft Maxwell) between experimental result and Ansoft Maxwell simulation in the region X = 10 to 11 micrometers, Z = 4 to 10 micrometers when V_{app} = +25 and -25 V. As mentioned in the manuscript, when the charged sTNP tip was brought closer to the top electrode, the difference between experimental and simulation results increased.
